# Four New Species of Jelly Fungi from Northeastern China

**DOI:** 10.3390/jof10070480

**Published:** 2024-07-12

**Authors:** Xia Wang, Tolgor Bau

**Affiliations:** 1 College of Mycology, Jilin Agricultural University, Changchun 130118, China; wangxia0115@163.com; 2 Key Laboratory of Edible Fungal Resources and Utilization (North), Ministry of Agriculture and Rural Affairs, Jilin Agricultural University, Changchun 130118, China

**Keywords:** Sirobasidiaceae, Dacrymycetaceae, phylogeny, taxonomy

## Abstract

Four new species of jelly fungi were described from northeastern China based on morphological and molecular evidence. These new species were classified into the four genera *Sirobasidium* (*Sirobasidium jilinense*), *Calocera* (*Calocera velutina*), *Dacrymyces* (*Dacrymyces jauensis*), and *Dacryopinax* (*Dacryopinax manghanensis*). Maximum likelihood and Bayesian analyses were performed using a combined nuc rDNA internal transcribed spacer region (ITS) and nuc 28S rDNA (nrLSU) dataset for the construction of phylogenetic trees. Morphological descriptions, line illustrations, and the ecological habits of these new species are provided.

## 1. Introduction

*Sirobasidium* Lagerh. & Pat. was established in 1892, with the type species being *Sirobasidium sanguineum* Lagerh. & Pat [1]. Due to the presence of hypobasidia that are divided vertically, the genus was first assigned to Tremellaceae Fr. In 1895, Möller proposed the establishment of Sirobasidiaceae Lindau due to the unique chain-like hypobasidia of this species [2]. Two years later, Sirobasidiaceae was officially published by Lindau [3]. Lowy then discovered that the French record of *S. cerasi* Bourdot & Galzin did not match the requirements of the genus concerning the size of its basidiomata, the quantity of chain-shaped basidia, and the size of its basidiospores. Lowy also provided a key for the recognized species *S. brefeldianum* Möller, *S. magnum* Boedijn, *S. albidum*, and *S. sanguineum* [4]. It was not until Bandoni examined the basidia and basidiospores of *Sirobasidium* that the spindle-shaped epiprobasidia shed by species in this genus were recognized as basidiospores [5]. In 2015, Roedel and Putzmann compiled a key for the *Sirobasidium* in their study of *S. albidum*, which included eight species [6]. Unfortunately, molecular information was not provided. At present, the genus contains ten species [3], three of which have been identified in China: *S. japonicum*, *S. magnum*, and *S. sanguineum* [7]. The chain-like hypobasidia and exfoliated epiprobasidia are the two primary characteristics of members of this genus. Phylogenetic analyses have shown the genus to be polyphyletic [8,9,10,11,12,13]. It is currently challenging to confirm the taxonomic status of *Sirobasidium*, however, as there are currently no molecular sequences available for the type species.

Dacrymycetaceae J. Schröt. (1888) belongs to Basidiomycota [3]. This family includes a variety of small-sized, morphologically diverse, and gelatinous species that are hard to find when dry. The majority of the basidiomata are yellow and contain carotene. The majority of basidiospores are often separated transversely, occasionally they can also be separated longitudinally or obliquely, or not septate when immature, septate by thin or thick walls; germination produces conidia or not; basidia and basidiospores contain oil droplets; hyphidia are present or absent; and with or without clamp connection [14].

In the 1960s and 1970s, McNabb conducted a detailed study on the genera of Dacrymycetaceae based on their morphology and compiled a key [15,16,17]. Since the 21st century, with the development of molecular systematics, researchers have conducted more in-depth research on Dacrymycetaceae species. In 2007, Shirouzu et al. found, in their phylogenetic analysis of *Dacrymyces* Nees based on 28S, that *Dacrymyces* was polyphyletic. While some species of *Dacrymyces* nest in Cerinomycetaceae Jülich or form a separate clade within Dacrymycetes, the majority of *Dacrymyces* species are found nested in Dacrymycetaceae [18]. In their 2009 study of Dacrymycetes in Japan, Shirouzu et al. described five new species and provided line illustrations [14]. Based on morphological observations, Malysheva (2013) identified the species of *Calocera* (Fr.) Fr. in Russia, described eight species of the genus, and provided line illustrations [19]. Later, Shirouzu et al. published eight new species of Dacrymycetes from New Zealand [20]. *Dacryopinax* G.W. Martin species from Brazil and Mexico were studied by Renato et al. and Castro-Santiuste et al., respectively [21,22]. The above studies show that most genera in Dacrymycetaceae are polyphyletic, and species are scattered in various branches of Dacrymycetaceae.

“Flora Fungorum Sinicorum Vol. 2 Tremellales et Dacrymycetales” by Liu was the primary focus of early Chinese research on Dacrymycetaceae species [7]. At the time, only morphological research was included due to the restricted conditions. Chinese researchers have recently studied species such as *Calocera* and *Dacrymyces* using multi-gene fragments [23,24,25]. However, most of the specimens used in these studies were collected from southern China, while there are relatively few specimens from northeastern China.

To enrich the species resources of jelly fungi in Northeast China. We have discovered four new species through morphological research and phylogenetic analysis. This study provided a detailed description and discussion of these species.

## 2. Materials and Methods

### 2.1. Specimen Collection

The specimens for this study were collected from Jilin Province and the Inner Mongolia Autonomous Region in China and deposited in the herbarium Fungarium of Jilin Agricultural University (FJAU).

### 2.2. Morphological Studies

Field records of fresh basidiomata, photographs of the habitat, and records after drying were used for macromorphological descriptions. The basidiomata’s color description was derived from Kornerup and Wanscher [26]. Using the technique of freehand slicing, utilizing water and 5% KOH solution as the floating carrier, and dyeing with 1% Phloxine B solution, the structures of basidia, basidiospores, hyphidia, and hyphae were examined under an optical microscope (Olympus CX33, Olympus, Tokyo, Japan). Twenty mature microstructures were chosen at random, examined under a microscope, and measured. “a–b × c–d” indicates the measurement of basidia, basidiospores, and hyphidia; “e–f” indicates the measurement of hyphae. Moreover, we determined the basidiospore’s Q value (Q = length/width). The extreme values of length, width, and diameter are denoted by the letters “a–b”, “c–d”, and “e–f”, respectively. For a given species, “n” denotes the ratio of the total number of measured basidiospores to the total number of measured specimens. All data were collected within a measurement range of 90%. The width was measured by choosing the area that was the widest. The apiculum was not taken into account when measuring the length of basidiospores. Along with the macroscopic morphology and microstructure features, a description and line charts are provided.

### 2.3. DNA Extraction, PCR and Sequencing

To extract DNA from dry specimens, the plant genome extraction kit from Kangwei Century (CWBIO, Beijing, China) was used. The ITS1F/ITS4 [27] and LR0R/LR7 [28,29] primers were used to amplify and sequence the ITS and nrLSU fragments, respectively. Sangon Bioengineering (Shanghai, China) Co., Ltd. was entrusted with the sequencing work, and the PCR reaction procedure was based on the method of Wang and Bau [30].

### 2.4. Molecular Phylogenetic Analyses

Sequencher 5.4.5 was used to observe the peak maps, further screen and sort the self-test sequences, and perform blast alignment [31]. ITS and nrLSU sequences were downloaded from GenBank based on the alignment outcomes and morphological similarity [32] (Table 1). The ITS and nrLSU sequences were aligned using the GINS-i algorithm with two iterative cycles only, using the online Mafft tool version 7 [33]. The alignment was manually adjusted and trimmed using MEGA 7.0.

The concatenated alignment of ITS (1–646) and nrLSU (647–1248) of *Sirobasidium* comprised 64 sequences. The concatenated alignment of ITS (1–682) and nrLSU (683–1517) of Dacrymycetaceae comprised 197 sequences. The best-fit model (edge-unlinked) was selected using the BIC criterion with ModelFinder [34]. The best-fit model of *Sirobasidium* and Dacrymycetaceae, according to BIC, was both SYM + I + G4. With the use of IQ-TREE [35] and the Shimodaira–Hasegawa-like approximate likelihood ratio test [36], maximum likelihood phylogenies were inferred under the edge–unlinked partition model for 10,000 ultrafast [37] bootstraps. The best-fit partition model (edge-unlinked) was again selected using the BIC criterion with ModelFinder. The best-fit model of *Sirobasidium* according to BIC was SYM + I + G4 (ITS) and K2P + I + G4 (nrLSU). The best-fit model of Dacrymycetaceae according to BIC was SYM + I + G4 (ITS) and GTR + F + I + G4 (nrLSU). Under the partition model, in which the first 25% of sampled data were discarded as burn-in, Bayesian inference phylogenies were inferred using MrBayes 3.2.6 [38]. Fig Tree v1.4.3 [39] and Photoshop 2021 (Adobe, Sam Jose, CA, USA) were then used to visualize the phylogenetic tree.

**Table 1:** Sequence information from phylogenetic trees.

Species	Locality	Sample No.	GenBank No.		References
			ITS	nrLSU	
*Bullera alba*	USA	CBS 501^T^	AF444368	AF075500	[40]
*Bullera hannae*	USA	CBS 8286^T^	AF444486	AF363661	[40]
*Bullera penniseticola*	USA	CBS 8623^T^	AF444471	AF363649	[40]
*Bullera unica*	USA	CBS 8290^T^	AF444441	AF075524	[40]
*Calocera bambusicola*	China	Wu9910 12	FJ195751	-	[23]
*Calocera cornea*	USA	AFTOL ID 438	AY789083	AY701526	[41]
*Calocera cornea*	Sweden	UPS F 940774	MN595626	MN595626	[41]
*Calocera cornea*	Germany	MW 55	-	AF291302	[42]
*Calocera cornea*	Canada	CBS 124 84	AB712437	AB472738	[43]
*Calocera furcata*	Russia	H Spirin 10949	MW191975	MW159088	[43]
*Calocera fusca*	New Zealand	PDD 107972	LC131406	LC131365	[20]
*Calocera glossoides*	Ukraine	CWU6247	MW191968	MW159084	[44]
*Calocera lutea*	New Zealand	PDD 107841	LC131413	LC131372	[20]
*Calocera palmata*	Japan	CBS 127 51	MH856777	MH868295	[32]
*Calocera pedicellata*	New Zealand	PDD 107830	LC131415	LC131374	[20]
***Calocera sinensis***	**China**	**FJAU68949**	**PP749224**	**-**	**This study**
*Calocera sinensis*	China	Dai22645	OL518939	OL518948	[32]
*Calocera tibetica*	China	Dai20171	MW549777	MW750403	
***Calocera velutina***	**China**	**FJAU68950^T^**	**PP776476**	**PP776564**	**This study**
***Calocera velutina***	**China**	**FJAU68951**	**PP776543**	**PP776565**	**This study**
*Calocera viscosa*	Japan	TUFC12873	AB712439	-	[43]
***Calocera viscosa***	**China**	**FJAU68917**	**-**	**PP749235**	**This study**
*Calocera viscosa*	Japan	175	-	AB299048	[18]
*Calocera viscosa*	Germany	AFTOL ID 1679^T^	DQ520102	DQ520102	[41]
*Calocera viscosa*	Sweden	UPS F 940773	MN595628	MN595628	[41]
*Cerinomyces cokeri*	Canada	TU135089	MW191983	MW191983	[44]
*Cerinomyces concretus*	Colombia	O F 919450^T^	MW191933	-	[44]
*Cerinomyces fugax*	USA	HHB 8856^T^	MW191905	MW159051	[44]
*Cerinomyces hesperidis*	USA	NY01782362^T^	MW191921	MW159065	[44]
*Cerinomyces inermis*	New Zealand	PDD87816^T^	MW191887	-	[44]
*Cerinomyces lipoferus*	Netherlands	ENZ20001	MZ147626	MZ147626	[44]
*Cerinomyces nepalensis*	Nepal	O F 904088	MW191896	-	[44]
*Cerinomyces neuhoffii*	Sweden	UPS F 941020^T^	MN595625	MN595625	[41]
*Cerinomyces pallidus*	USA	ARIZ AN09245	MZ147624	-	[44]
*Cerinomyces paulistanus*	Brazil	O Ryvarden 24759^T^	MW191934	-	[44]
*Cerinomyces pinguis*	Nepal	O F 904085^T^	MW191907	MW159043	[44]
*Cerinomyces tortus*	Sweden	UPS F 946515	MW191999	MW191999	[44]
*Cerinomyces volaticus*	Russia	LE295748	MW191901	MW159047	[44]
*Dacrymyces adpressus*	Japan	554	-	AB472729	[14]
*Dacrymyces ancyleus*	Japan	MAFF 241177^T^	AB712448	AB472713	[43]
***Dacrymyces capitatus***	**China**	**FJAU68952**	**PP749225**	**PP749236**	**This study**
*Dacrymyces cerebriformis*	China	Dai 19832^T^	OM955202	OM955197	[25]
*Dacrymyces chiangraiensis*	Thailand	MFLU 16 0572	KY498587	-	[45]
*Dacrymyces chrysocomus*	Japan	CBS 280 84	AB712451	AB712427	[43]
***Dacrymyces chrysospermus***	**China**	**FJAU68956**	**PP749226**	**PP749237**	**This study**
*Dacrymyces citrinus*	New Zealand	PDD 107915^T^	LC131417	LC131376	[20]
*Dacrymyces corticioides*	USA	NY02686162	MW191944	MW159068	[44]
*Dacrymyces cylindricus*	New Zealand	PDD 105052^T^	LC131419	LC131378	[20]
*Dacrymyces cyrtosporus*	New Zealand	PDD 107980^T^	LC131422	LC131381	[20]
*Dacrymyces dictyosporus*	Japan	HHB 8618	AB712454	AB712429	[43]
*Dacrymyces fennicus*	Finland	H Miettinen 21174	MW191957	MW159071	[44]
*Dacrymyces flabelliformis*	New Zealand	HHB 18308^T^	AB712455	AB712430	[43]
*Dacrymyces grandinioides*	Kenya	K M 237139^T^	MW191980	MW191980	[44]
*Dacrymyces intermedius*	New Zealand	PDD 107851	-	LC131384	[20]
*Dacrymyces invisibilis*	Chile	14597MD^T^	MH230100	MH230102	[46]
***Dacrymyces jauensis***	**China**	**FJAU68959**	**PP776532**	**PP776577**	**This study**
***Dacrymyces jauensis***	**China**	**FJAU68960**	**PP776533**	**PP776578**	**This study**
***Dacrymyces jauensis***	**China**	**FJAU68961^T^**	**PP776534**	**PP776579**	**This study**
*Dacrymyces lacrymalis*	Japan	TUFC13327	AB712456	AB299069	[43]
*Dacrymyces longistipitatus*	New Zealand	PDD 107997^T^	LC131426	LC131387	[20]
*Dacrymyces microsporus*	China	Wu561	AB712458	OL546779	[32]
*Dacrymyces minor*	Japan	TUFC12836	AB712458	-	[43]
*Dacrymyces minutus*	Japan	648	AB712459	AB472733	[43]
*Dacrymyces novae-zelandiae*	New Zealand	PDD 107953	LC131428	LC131391	[20]
*Dacrymyces ovisporus*	Norway	H Spirin 11145	MW191960	MW159073	[44]
*Dacrymyces pachysporus*	New Zealand	PDD 105004^T^	LC131429	LC131392	[20]
*Dacrymyces parastenosporus*	New Zealand	PDD 104963^T^	LC131432	LC131395	[20]
*Dacrymyces pezizoides*	Japan	TNS F 54909	LC386890	LC386894	[47]
*Dacrymyces pinacearum*	Japan	UPS F 593533	MN595637	MN595637	[41]
***Dacrymyces puniceus***	**China**	**FJAU68955**	**PP749227**	**PP749238**	**This study**
*Dacrymyces san-augustinii*	China	Wu544	OL587813	OL546781	[32]
*Dacrymyces sinostenosporus*	China	Dai20003^T^	MW540888	MW540890	[25]
*Dacrymyces stenosporus*	New Zealand	PDD 105018^T^	LC131433	LC131396	[20]
*Dacrymyces stillatus*	Sweden	UPS F 939814 1	MN595677	MN595677	[41]
*Dacrymyces subalpinus*	Japan	228	-	AB299060	[18]
*Dacrymyces subantarcticensis*	New Zealand	PDD 107948	LC131435	LC131399	[20]
*Dacrymyces subarcticus*	Japan	HNo 544	AB712467	-	[43]
*Dacrymyces variisporus*	Japan	TUFC14203	AB712470	-	[43]
*Dacrymyces venustus*	Ethiopia	O Adane 150	MW191949	MW159075	[44]
*Dacryomitra pusilla*	Sweden	UPS F 176774	MN595639	MN595639	[41]
*Dacryopinax elegans*	USA	HHB 18731	AB712471	AB712433	[43]
*Dacryopinax indacocheae*	Venezuela	CRM 72	AB712472	AB712434	[43]
*Dacryopinax lowyi*	Mexico	27618	MN733712	MN733723	[22]
***Dacryopinax manghanensis***	**China**	**FJAU68940**	**PP749230**	**PP749241**	**This study**
***Dacryopinax manghanensis***	**China**	**FJAU68938**	**PP749228**	**PP749239**	**This study**
***Dacryopinax manghanensis***	**China**	**FJAU68941**	**PP749231**	**PP749242**	**This study**
***Dacryopinax manghanensis***	**China**	**FJAU68943^T^**	**PP749233**	**PP749244**	**This study**
***Dacryopinax manghanensis***	**China**	**FJAU68942**	**PP749232**	**PP749243**	**This study**
***Dacryopinax manghanensis***	**China**	**FJAU68939**	**PP749229**	**PP749240**	**This study**
*Dacryopinax martinii*	Mexico	682	MN733715	MN733726	[22]
*Dacryopinax maxidorii*	Brazil	RLMA306	OK257531	OK257536	[48]
*Dacryopinax primogenitus*	Costa Rica	MIN 862738	KT251038	KT251039	[49]
*Dacryopinax* sp.	Kenya	H7008759	MW191959	MW159091	[44]
***Dacryopinax spathularia***	**China**	**FJAU68908**	**PP749234**	**PP749245**	**This study**
*Dacryopinax spathularia*	USA	H Miettinen 16740	MW191973	MW159085	[44]
*Dacryopinax spathularia*	Indonesia	H Miettinen 20559	MW191976	MW159092	[44]
*Dacryopinax spathularia*	Chinna	Wu 331	OL614833	OL616184	[32]
*Dacryopinax sphenocarpa*	Japan	534	-	AB472726	[14]
*Dendrodacrys brasiliense*	Brazil	INPA 241458^T^	AB744230	AB723514	[50]
*Dendrodacrys ciprense*	Cyprus	UPS F 946590^T^	OM519385	OM519385	[51]
*Dendrodacrys concrescens*	Sweden	UPS F 946602^T^	OM519390	OM519390	[51]
*Dendrodacrys dendrocalami*	Japan	TUFC13914	AB712453	AB712428	[43]
*Dendrodacrys ellipsosporum*	Spain	UPS F 946604^T^	OM519392	OM519392	[51]
*Dendrodacrys kennedyae*	Panama	TAAM192134^T^	OP529836	OP529830	[51]
*Dendrodacrys laetum*	Kenya	H7008757^T^	OP529841	OP529841	[51]
*Dendrodacrys oblongisporum*	Norway	UPS F 946599	OM519399	OM519399	[51]
*Dendrodacrys rigoratum*	Japan	TUFC12845^T^	AB712447	-	[14]
*Ditiola haasii*	Czech Republic	PRM 944647	MW557322	MW557322	[52]
*Ditiola peziziformis*	Finland	H Haikonen 2426	MW191972	MW159070	[18]
*Fellomyces horovitziae*	USA	CBS 7515^T^	-	AF189856	[53]
*Femsjonia monospora*	Thailand	MFLU 16 0608	KY498588	-	[45]
*Femsjonia uniseptata*	Japan	TNS F 54019^T^	NR_164095	NG_060435	[20]
*Fibulobasidium inconspicuum*	USA	CBS 8237^T^	AF444318	AF363641	[40]
*Fibulobasidium murrhardtense*	China	CBS 9109^T^	GU327540	AF416648	[54]
*Fibulobasidium sirobasidioides*	Germany	RJB 12787	-	AF416644	[55]
*Fonsecazyma mujuensis*	China	CBS 10308^T^	KF036595	DQ333884	[12]
*Genolevuria amylolytica*	China	CBS 10048^T^	KF036585	AY562134	[12]
*Genolevuria armeniaca*	China	CBS 10050	KF036587	AY562140	[12]
*Genolevuria bromeliarum*	Brazil	BI20	EU386359	DQ784566	[56]
*Genolevuria tibetensis*	China	AS 2 2653^T^	EF363146	EF363143	[57]
*Guepiniopsis alpina*	USA	HAY F 002724	OR767855	-	[32]
*Guepiniopsis buccina*	USA	AFTOL ID 888	DQ206986	AY745711	[41]
*Guepiniopsis estonica*	Sweden	UPS F 940137	MN595632	MN595632	[41]
*Heterotextus miltinus*	New Zealand	PDD 107924	LC131438	LC131402	[20]
*Kockovaella imperatae*	USA	CBS 7554^T^	AB054091	AF189862	[53]
*Pseudotremella allantoinivorans*	USA	CBS 9604^T^	AY315664	AY315662	[58]
*Pseudotremella moriformis*	USA	CBS 7810	AF444331	AF075493	[40]
*Sirobasidium apiculatum*	Japan	MY62 1	LC203425	LC203426	[13]
*Sirobasidium apiculatum*	Japan	MY62 4	LC203428	LC203429	[13]
*Sirobasidium brefeldianum*	Spain	AM71	JN053472	JN043578	[9]
*Sirobasidium brefeldianum*	USA	CBS 7805	AF444330	AF075492	[40]
*Sirobasidium japonicum*	Japan	MY111 05	LC203420	LC016573	[13]
*Sirobasidium japonicum*	Japan	MY111 09	LC203422	LC203423	[13]
***Sirobasidium jilinense***	**China**	**FJAU68667**	**PP749214**	**-**	**This study**
***Sirobasidium jilinense***	**China**	**FJAU68670^T^**	**PP749217**	**PP749222**	**This study**
***Sirobasidium jilinense***	**China**	**FJAU68668**	**PP749215**	**PP749220**	**This study**
***Sirobasidium jilinense***	**China**	**FJAU68669**	**PP749216**	**PP749221**	**This study**
***Sirobasidium jilinense***	**China**	**FJAU68672**	**PP749219**	**-**	**This study**
***Sirobasidium jilinense***	**China**	**FJAU68671**	**PP749218**	**PP749223**	**This study**
*Sirobasidium magnum*	Spain	AM70	JN053497	JN043603	[9]
*Sirobasidium magnum*	Netherlands	CBS 6803	AF444314	KY109657	[40]
*Sirobasidium magnum*	Netherlands	CBS 6964	KY105421	KY109658	[59]
***Sirobasidium magnum***	**China**	**FJAU68996**	**PP741731**	**PP741733**	**This study**
***Sirobasidium magnum***	**China**	**FJAU68997**	**PP741732**	**PP741734**	**This study**
*Tremella giraffa*	Germany	CCJ 1553	AF042453	AF042271	[60]

Note: “-” denotes the absence of pertinent genetic data. Sequences newly generated in this study are indicated in bold. The type specimens are those designated with a “T”.

## 3. Results

### 3.1. Phylogenetic Analyses

**Figure 1:**
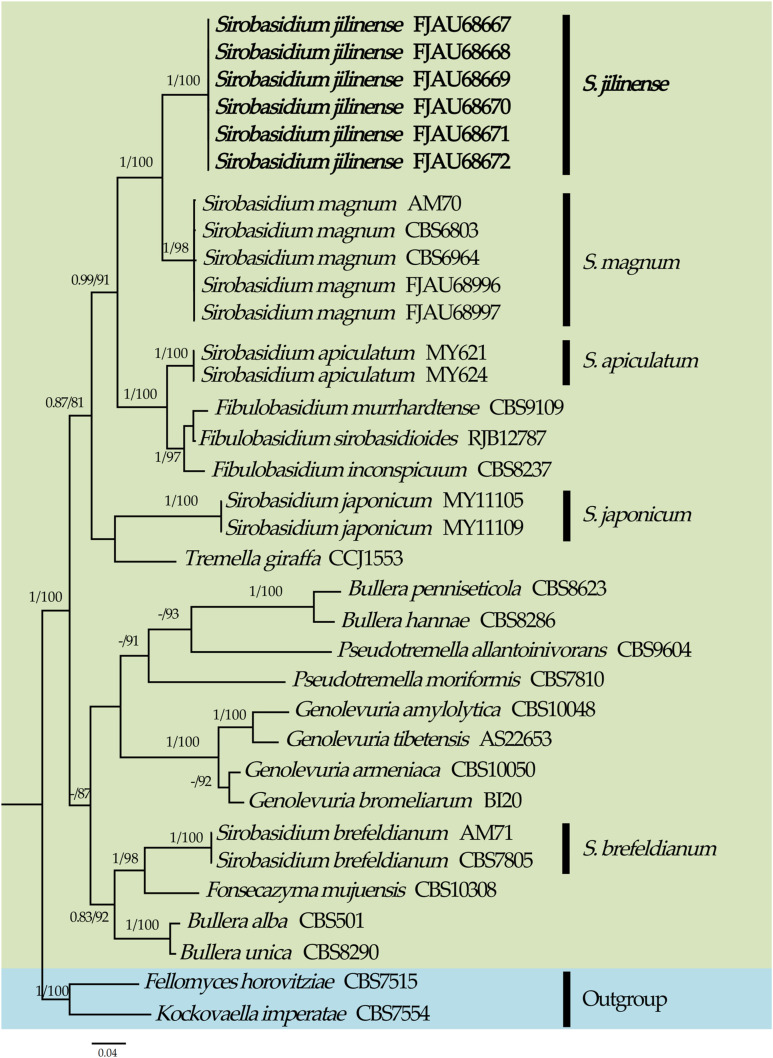
Phylogeny of *Sirobasidium* and related species by Bayesian inference based on the ITS + nrLSU dataset. New species are indicated in bold.

**Figure 2:**
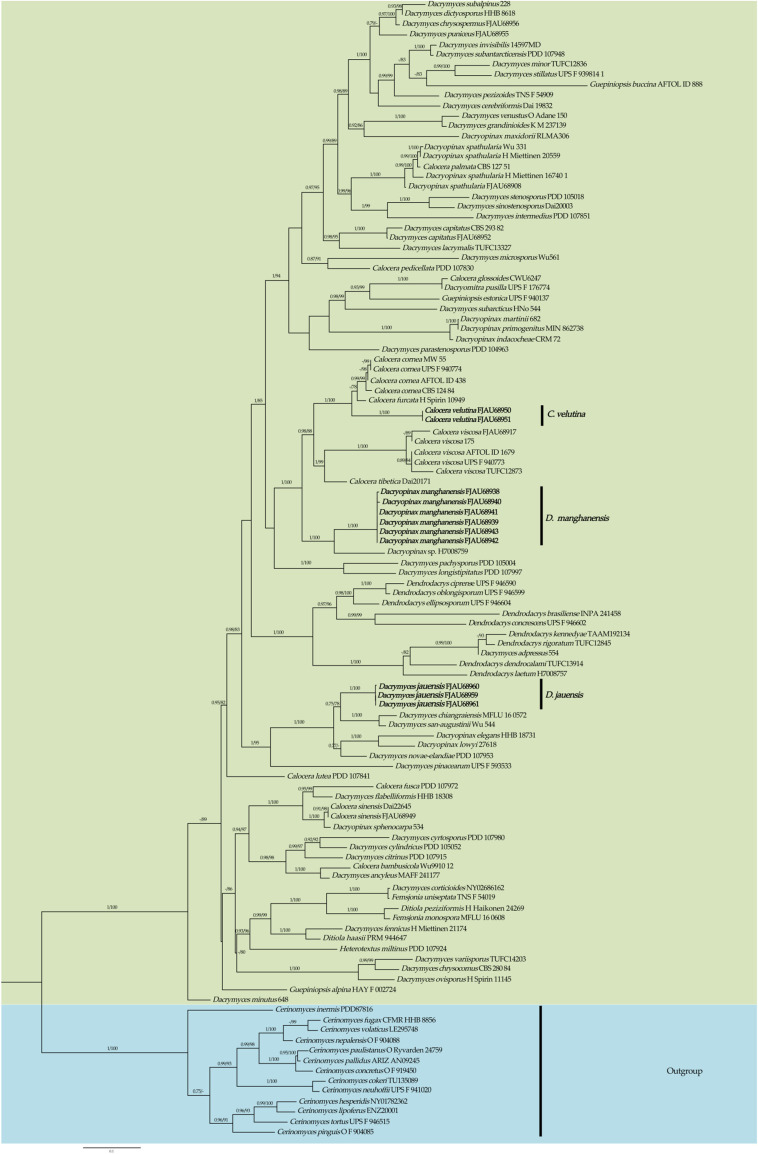
Phylogeny of Dacrymycetaceae by Bayesian inference based on the ITS + nrLSU dataset. New species are indicated in bold.

This study generated a total of 46 new sequences, including 24 ITS sequences and 22 nrLSU sequences. These new sequences have been uploaded to GenBank. The multi-locus dataset (ITS + nrLSU) of *Sirobasidium* had an aligned length of 1248 total characters), including gaps. The multi-locus dataset (ITS + nrLSU) of Dacrymycetaceae had an aligned length of 1517 total characters, including gaps. Only the topological structures of Bayesian inference are displayed, as the topological structures of ML and BI are very similar. Bootstrap support (BS) values ≥ 75% and Bayesian posterior probability (PP) values ≥ 0.75 are indicated on branches (BS/PP) (Figure 1 and Figure 2).

From the phylogenetic tree of *Sirobasidium* and its related species constructed with *Fellomyces horovitziae* Spaaij, G. Weber & Oberw. and *Kockovaella imperatae* Nakase, I. Banno & Y. Yamada as outgroups (highlighted in blue), it can be seen that *Sirobasidium* is polyphyletic (Figure 1). High support rates (PP = 1.00, MLbs = 100%) were attained by the six specimens of the new species *Sirobasidium jilinense* T. Bau et X. Wang sp. nov., which were grouped in an independent branch. *S. jilinense* and *S. magnum* share a sister relationship and received high support (PP = 1.00, MLbs = 100%). The novel species could also be easily distinguished from other species, thanks to its distinct placement in the phylogenetic tree (Figure 1).

From the phylogenetic tree of Dacrymycetaceae constructed with Cerinomycetaceae as an outgroup (highlighted in blue), it can be seen that *Dacrymyces*, *Dacryopinax*, and *Calocera* are polyphyletic. The species belonging to each genus are dispersed across different branches of Dacrymycetaceae (Figure 2).

The two specimens of the recently discovered species *Calocera velutina* T. Bau et X. Wang sp. nov. were grouped into a separate branch and had a high support rate (PP = 1.00, MLbs = 100%). *C. velutina* forms a sister clade with *C. cornea* (Batsch) Fr. and *C. furcata* (Fr.) Fr., which is highly supported (PP = 1.00, MLbs = 100%). At the same time, the new species can be distinguished from other species in the genus in the phylogenetic tree (Figure 2).

A high support rate (PP = 1.00, MLbs = 100%) was obtained for the three specimens of the new species, *Dacrymyces jauensis* T. Bau et X. Wang sp. nov. They were grouped into an independent branch. *D. jauensis* forms a sister relationship with the clade consisting of *D. chiangraiensis* Ekanayaka, Karun., Q. Zhao & KD Hyde, and *D. san-augustinian* Kobayasi and gained a lower support rate (PP = 0.75, MLbs = 78%). This suggests that other species among them have not yet been identified. The new species and other species could also be well distinguished in the phylogenetic tree (Figure 2).

The six specimens of the new species *Dacryopinax manghanensis* T. Bau et X. Wang sp. nov. were clustered into an independent branch and obtained a high support rate (PP = 1.00, MLbs = 100%). It is a sister clade to *Dacryopinax* sp. (H7008759), with a high support rate (PP = 1.00, MLbs = 100%), and which is well distinguished from other species.

### 3.2. Taxonomy

**Figure 3:**
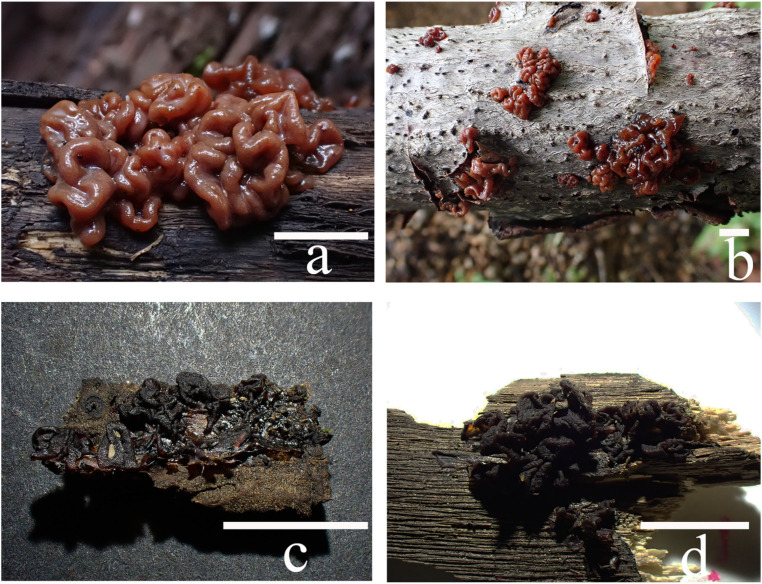
Basidiomata of *Sirobasidium jilinense*: (**a**) fresh (FJAU68670); (**b**) fresh (FJAU68668); (**c**,**d**) dry (FJAU68667). Scale bars: (**a**,**b**) = 1 cm; (**c**,**d**) = 0.5 cm.

**Figure 4:**
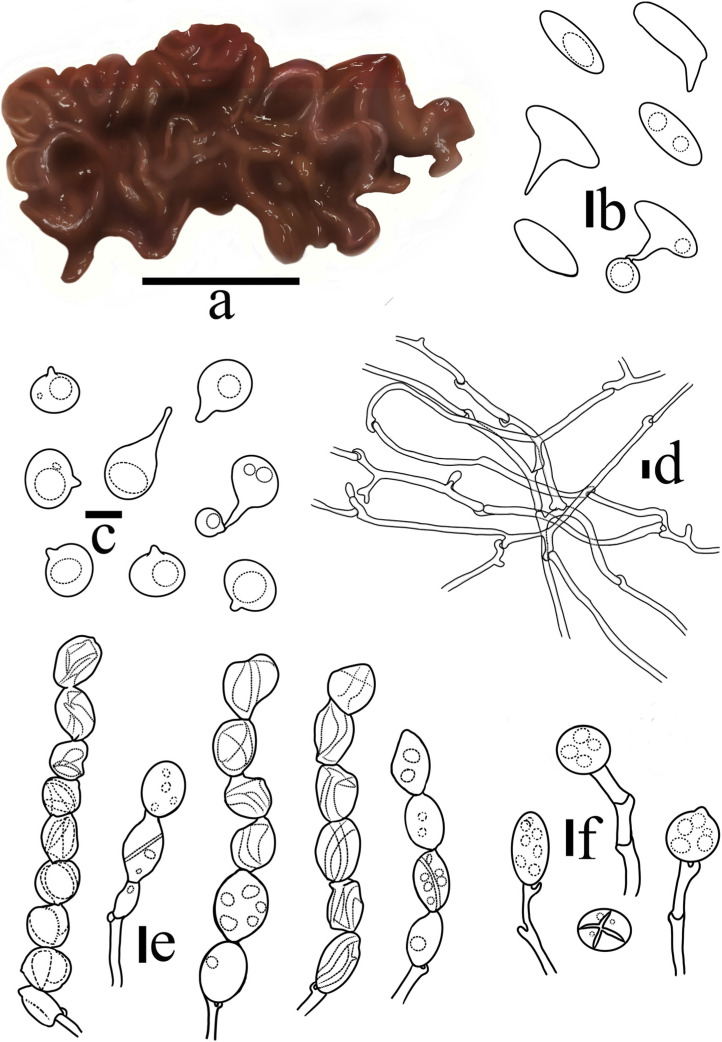
*Sirobasidium jilinense* T. Bau et X. Wang, sp. nov. (**a**) Basidiomata; (**b**) Epibasidia; (**c**) Basidiospores; (**d**) Hyphae; (**e**) Hypobasidia; (**f**) Probasidia. (Scale bars: (**a**) = 1 cm; (**b**,**e**,**f**) = 10 μm; (**c**,**d**) = 5 μm).

***Sirobasidium jilinense*** T. Bau et X. Wang, **sp. nov.** (Figure 3 and Figure 4)

**MycoBank number:** 853841

**Diagnosis:** *Sirobasidium jilinense* is characterized by cerebriform basidiomata, which are reddish brown to dark reddish brown when fresh and dark brown when dry. The hypobasidia consist of three to nine chains and are divided vertically or obliquely into four cells, whereas the basidiospores are subglobose to broadly ellipsoid, and the hyphae are thick-walled.

**Etymology:** “jilinense” refers to the discovery of a type specimen in Jilin Province, China.

**Type:** CHINA. Jilin Province, Jilin, Jiaohe, Shansong Ridge, on rotten wood of *Quercus*, elev. 577.2 m, 43°75′ N, 127°33′ E, 26 August 2023, T. Bau and Xia Wang, (FJAU68870, holotypus!). Same location, 29 July 2023, Xia Wang, (FJAU68668, paratypus!).

Basidiomata are soft gelatinous when fresh, easily rotting, cerebriform, reddish brown (8E5–E8) to dark reddish brown (8F5–F8), usually remaining coalescing, occasionally separate, 1–3 cm long and 1 cm thick. The hymenium surface is smooth, with full edges, folded, and relatively blunt. Shrinkage when dry, dark brown (8F1–F5), fragile.

Cross section without medulla, composed of the hymenium and intertwined jelly hyphae. Hymenium peripheral, pale yellow, composed of probasidia and hypobasidia. Probasidia 12–20 × 11–16 μm, globose to ellipsoid, light yellow, containing oil droplet-like substances, with clamp connection at the base; hypobasidia, 14–22 × 10–15 μm, ellipsoid to ovoid, the vertical or diagonal partitions are 4-cells, 3–9 chain-like structures, wrinkled at maturity, light yellow, containing oil droplet-like substances, with clamp connection at the base; epibasidia, 13–18 × 5–8 μm, spindle-shaped, detached, containing oil droplet-like substances. Basidiospores 6.7–9.4 × 6.7–7.2 μm, Q = 1.04–1.37 (n = 100/5), subglobose to broadly ellipsoid, apiculate, colorless, smooth, with oil droplets, germinating to produce regenerated spores or germination tubes. Hyphae with rich clamp connection, thick-walled, 2.3–3.6 µm in diameter.

**Habitat:** In summer and autumn, it grows on fallen trees or branches such as *Acer* and *Quercus* in broad-leaved forests.

**Distribution:** Currently, only known in the Jilin Province, China, Asia.

**Additional specimens examined:** China. Jilin Province: Jilin, Jiaohe, Shansong Ridge, on fallen branches of *Acer*, July 24, 2022, Liyang Zhu, FJAU68667 (Z22072428). Same location, Shien Wang, FJAU68669 (E2307209); Jilin, Huandian, Hongshi National Forest Park, on fallen branches of *Acer*, August 27, 2023, T. Bau & Xia Wang FJAU68671 (W23082706), T. Bau & Mu Liu, FJAU68672 (lm230890).

**Figure 5:**
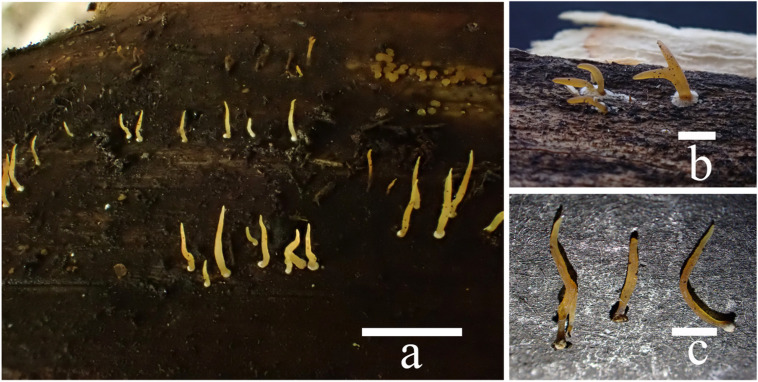
Basidiomata of *Calocera velutina*: (**a**) fresh (FJAU68950); (**b**,**c**) dry (FJAU68951, FJAU68950). Scale bars: (**a**–**c**) = 5 mm.

**Figure 6:**
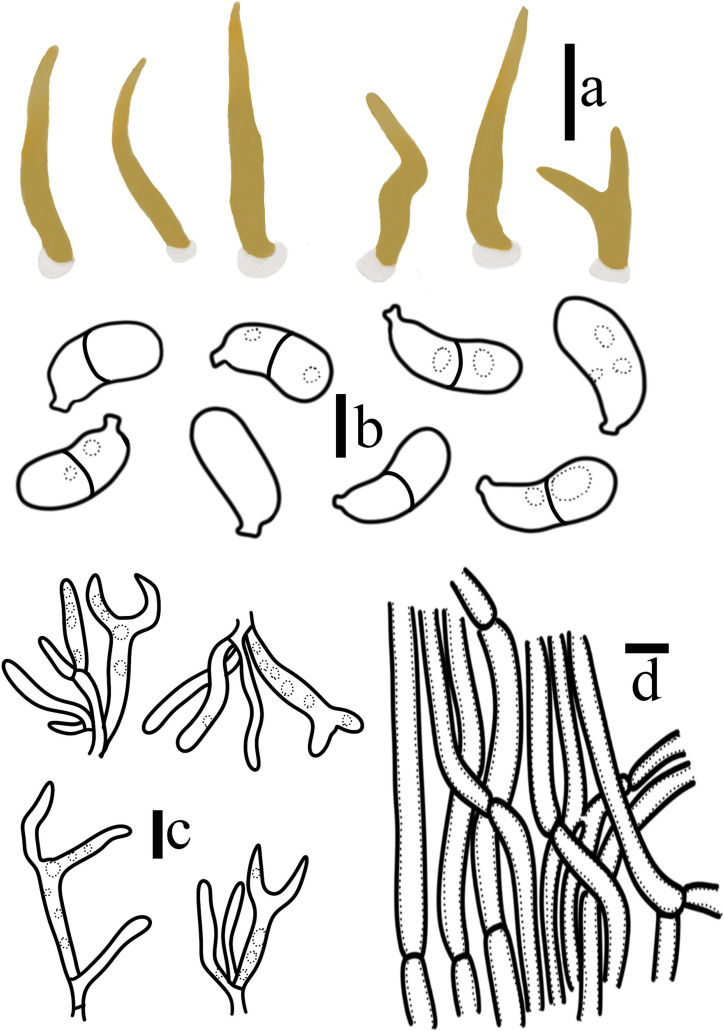
*Calocera velutina* T. Bau et X. Wang, sp. nov. (**a**) Basidiomata; (**b**) Basidiospores; (**c**) Basidia and hyphidia; (**d**) Central hyphae. (Scale bars: (**a**) = 1 cm; (**b**) = 5 μm; (**c**,**d**) = 10 μm).

***Calocera velutina*** T. Bau et X. Wang, **sp. nov.** (Figure 5 and Figure 6)

**MycoBank number:** 854036

**Diagnosis:** *Calocera velutina* is characterized by stipitate and pileate when young, needle-like when mature, light yellow, with a white disc-shaped villus at the base, simple or dichotomously branched. Hyphae thick-walled, without clamp connection. In summer, they grow on decaying trees in coniferous forests.

**Etymology:** “velutina” refers to the base of the basidiomata with villus.

**Type:** CHINA. Jilin Province, Yanbian Korean Autonomous Prefecture, Antu County, Erdaobaihe Town, Forest Industry Cultural Park, on rotten wood of *Pinus* L., elev. 708 m, 42°43′ N, 128°12′ E, 31 July 2022, T. Bau and Xia Wang, (FJAU68950, holotypus!). Jilin Province, Huadian City, Hongshi National Forest Park, 27 August 2023, T. Bau and Mu Liu, (FJAU68951, paratypus!).

Basidiomata are gelatinous when fresh, stipitate and pileate when young, needle-like when mature, light yellow (4A4–A5), slightly lighter at the tip, with white (4A1) disc-shaped villus at the base, simple or dichotomously branched, up to 15 mm high. The hymenium is located in the upper middle part, and the base is sterile. When dry, it is greyish yellow (4B3–B6, 4C5), and the surface under the body mirror is fine and granular.

The transverse section of the apex of the basidiomata is composed of the hymenium and interwoven hyphae. The transverse section of the basidiomata cylinder presents three annular zones. The central hyphae are densely arranged vertically, surrounded by loosely interlaced hyphae, and the periphery is the hymenium. Hymenium amphigenous is composed of probasidia, basidia, and hyphidia. Probasidia 18–25 × 2.9–4.4 μm, sub-clavate, forked when mature, containing oil droplet-like substances, with septate at the base; basidia 21–33 × 3.5–4.8 μm, clavate, containing oil droplet-like substances, with septate at the base; sterigmata 10–17 × 2.3–3.7 μm, cylindrical, the top gradually becomes pointed and gradually weakens when mature, containing oil droplet-like substances. Hyphidia 19–28 × 1.8–2.7 μm, narrowly cylindrical, simple or slightly curved, with septum at the base. Basidiospores 9.1–10.4 × 4.2–5.3 μm, Q = 1.90–2.27 (n = 40/2), curved cylindrical, narrow top, smooth, thin-walled, mature 1 septate, septate thin-walled, contains oil droplet-like substances. No germination was observed. Central hyphae 4.9–6.8 μm in diameter, septate, slightly rough, thick-walled. Interlaced hyphae, 3.4–5.3 μm in diameter, septate, thick-walled, basal hyphae 2.5–3.8 μm in diameter. All structures without clamp connection.

**Habitat:** In summer, it grows on decaying trees in coniferous forests.

**Distribution:** Currently, only known in the Jilin Province, China, Asia.

**Figure 7:**
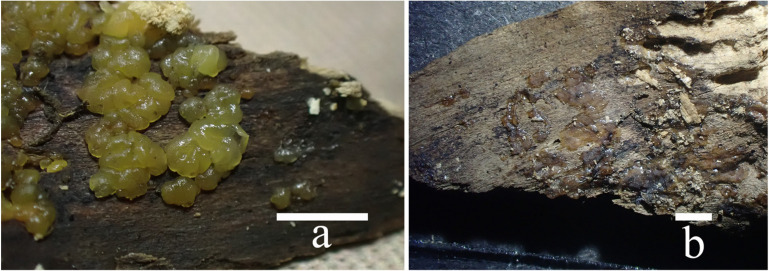
Basidiomata of *Dacrymyces jauensis*: (**a**) fresh (FJAU68961); (**b**) dry (FJAU68961). Scale bars: (**a**) = 1 cm, (**b**) = 0.5 cm.

**Figure 8:**
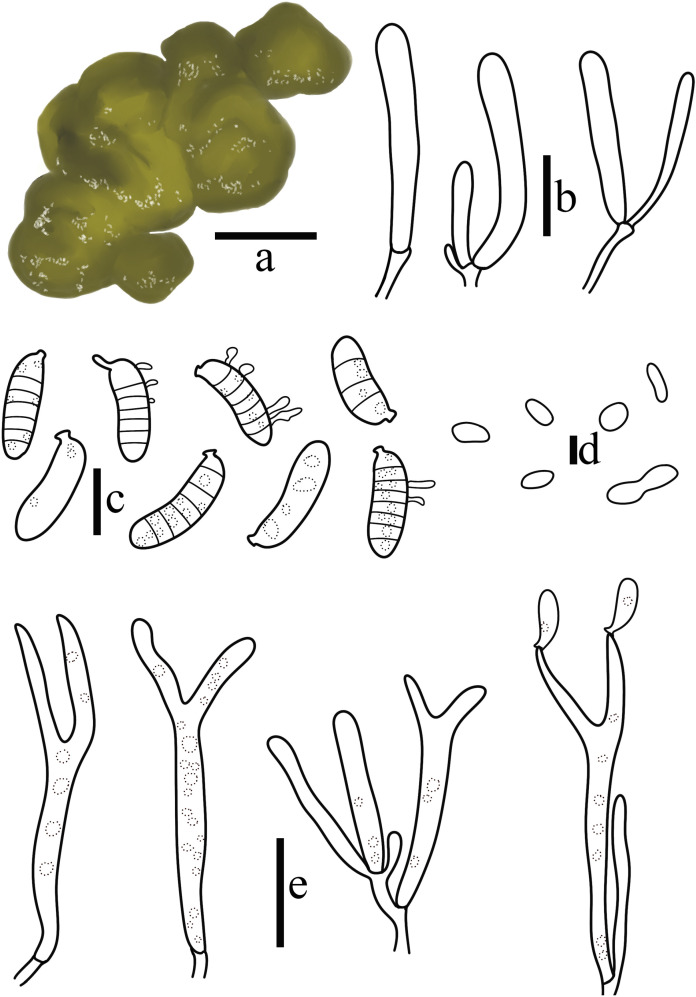
*Dacrymyces jauensis* T. Bau et X. Wang, sp. nov. (**a**) Basidiomata; (**b**) Probasidia; (**c**) Basidiospores; (**d**) Conidia; (**e**) Basidia. (Scale bars: (**a**) = 1 cm; (**b**–**d**) = 10 μm; (**e**) = 20 μm).

***Dacrymyces jauensis*** T. Bau et X. Wang, **sp. nov.** (Figure 7 and Figure 8)

**MycoBank number:** 854037

**Diagnosis:** *Dacrymyces jauensis* is characterized by basidiomata flat to cushion-shaped when young, cinnamon, golden yellow to brownish yellow; basidiospores sausage-shaped, separated horizontally when mature, usually divided into 7 septate; germination produces rod-shaped conidia; hyphidia absent.

**Etymology:** “jauensis” refers to the discovery of a type specimen at Jilin Agricultural University.

**Type:** CHINA. Jilin Province, Changchun city, wild plantation garden of the campus of Jilin Agricultural University, grows on highly decayed wood of broad-leaved trees in broad-leaved forests, 43°81′ N, 125°40′ E, 235 m, 16 July 2022, Xia Wang & Tolgor Bau, (FJAU68961, 22071605W, holotypus!). Same location, 4 July 2022, Xia Wang & Tolgor Bau, (FJAU68959, T22070437W; FJAU68960, T22070438W, paratypus!).

Basidiomata are soft gelatinous when fresh, flat to cushion-shaped when young, cinnamon, golden yellow (4C6), up to 0.5 cm, usually remaining coalescing when mature, brownish yellow (5D6, 5E5), sessile, up to 2.5 cm. Hymenium surface with wrinkles and grooves, edge curved, folded, wavy, blunt. When dry, it shrinks and collapses in a brown (6E6), making it difficult to observe.

The transverse section is composed of hymenium and context hyphae. Hymenium amphigenous. Probasidia 32–47 × 3.7–6.3 μm, cylindrical, subclavate to broadly clavate, thin-walled, transparent, forked when mature, containing oil-dripping substance, basally septate; sterigmata 11–33 × 3.0–4.4 μm, cylindrical, apically acuminate when mature, gradually weakening. Hyphidia absent. Basidiospores 15.9–20.9 × 5.1–6.3 μm, Q = 2.80–3.49 (n = 90/3), sausage-shaped, smooth, thin-walled, transparent, separated horizontally when mature, usually divided into 7 septate, occasionally 0–3 septate, thin-walled, containing oil drop-like substance, and conidia germinate at the septate. Conidia 4.8–12.3 × 1.4–1.8 μm, long cylindrical to rod-shaped. Context hyphae 1.6–3.2 μm in diameter, thin-walled, septate, septate not swollen. All structures without clamp connection.

**Habitat:** In summer, it grows on highly decayed wood of broad-leaved trees in broad-leaved forests.

**Distribution:** Currently, only known in the Jilin Province, China, Asia.

**Figure 9:**
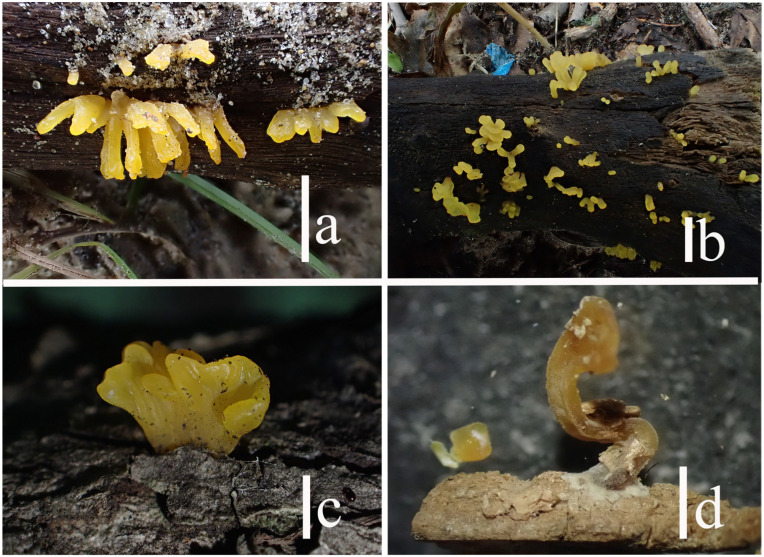
Basidiomata of *Dacryopinax manghanensis*: (**a**) fresh (FJAU68943); (**b**) fresh (FJAU68940); (**c**) fresh (FJAU68941); (**d**) dry (FJAU68938). Scale bars: ((**a**–**c**) = 0.5 cm; (**d**) = 0.2 cm).

**Figure 10:**
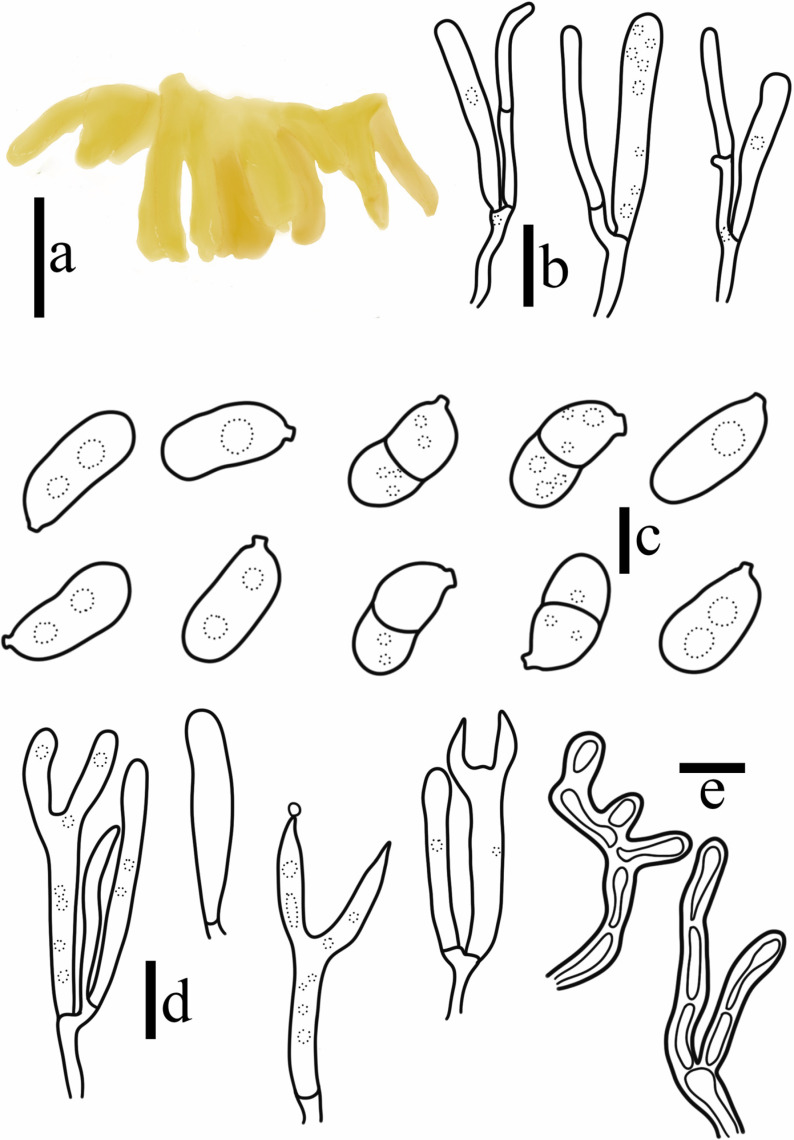
*Dacryopinax manghanensis* T. Bau et X. Wang. (**a**) Basidiomata; (**b**) Probasidia and hyphidia; (**c**) Basidiospores; (**d**) Basidia; (**e**) Marginal hyphae. ((**a**) = 0.5 cm; (**b**) = 5 μm; (**c**) = 2 μm; (**d**,**e**) = 10 μm).

***Dacryopinax manghanensis*** T. Bau et X. Wang, **sp. nov.** (Figure 9 and Figure 10)

**MycoBank number:** 853843

**Diagnosis:** *Dacryopinax manghanensis* is characterized by small basidiomata, 1–5 mm wide, 1–8 mm high, spathulate and stipitate to substipitate, light yellow, cross section without medulla, the short and fine villi on the sterile surface. Basidiospores curved cylindrical to navicular, with 0–1 septate at maturity, thin-walled septate, and no germination observed; cortical hyphae curved, thick-walled, swollen, and branched.

**Etymology:** “manghanensis” is a Mongolian word for “sandy land”, which means that type specimens are collected from rotten wood in sandy land.

**Type:** CHINA. Inner Mongolia Autonomous Region, Tongliao, in the later stage of the left wing of Horqin, Wudantara Forest Farm, fallen branches of broad-leaved trees, 43°01′ N, 122°44′ E, 363 m, 16 July 2022, Weinan Hou & Tolgor Bau, (FJAU68943, H2207159, holotypus!). Same location, 15 July 2023, Hong Cheng & Tolgor Bau, (FJAU68940, C2371524, paratypus!).

When the basidiomata are fresh, soft gelatinous, spathulate, and stipitate to substipitate when young, the stipitate is light yellow (4A4–A5) and slightly hyaline, petaloid when mature, surface with longitudinal ridges, 1–5 mm wide, and 1-8 mm high. Hymenial surface smooth, with entire edges, blunt. The short and fine villi on the sterile surface are visible under a stereomicroscope. When dry, it shrinks, with a white (4A1) base, a dark yellow (4C8) surface on the hymenial, and obvious longitudinal edges on the stipitate.

Cross section without medulla, composed of the hymenium, context hyphae, and cortical hyphae. Hymenium unilateral, occasionally bilateral, composed of basidia and hyphidia. Probasidia 18–27 × 2.5–3.5 μm, cylindrical to clavate, thin-walled, hyaline, forked when mature, containing oil droplet-like substances, with septate at the base; sterigmata 7–13 × 1.8–2.9 μm, the top gradually becomes pointed and gradually weakens when mature. Hyphidia 15–27 × 1.4–2.2 μm, narrow cylindrical, thin-walled, hyaline, simple or slightly curved, with 1 septum at the base. Basidiospores 7.9–9.0 × 3.7–4.9 μm, Q = 1.73–2.07 (n = 80/4), curved cylindrical to navicular, apiculate, smooth, thin-walled, hyaline, mature 0–1 septate, septate thin-walled, contains oil droplet-like substances. No germination was observed. Cortical hyphae 4.5 μm in diameter, cylindrical branching at the end, curved, septum, constriction at intervals, thick-walled. All structures without clamp connection.

**Habitat:** In summer and autumn, it decays on fallen branches in broad-leaved forests or mixed coniferous and broad-leaved forests.

**Distribution:** Currently, it is only distributed in China, Inner Mongolia Autonomous Region, and Jilin Province.

**Additional specimens examined:** CHINA. Jilin Province, Tonghua, Yuhuangshan Park, broad-leaved forest rotting wood, 14 August 2022, Xia Wang, FJAU68941 (2281405W); Changchun, Jilin Agricultural University Campus Back Mountain, 16 September 2022, Xia Wang, FJAU68942 (2291601W); Tonghua, Yushan Park in Ji’an City, 6 July 2023, Zhengqing Chen, FJAU68938 (Q237603); Changchun, Jingyue Pool National Forest Park, 12 July 2023, Xia Wang, FJAU68939 (W23071206).

## 4. Discussion

*Sirobasidium jilinense* is easily confused with the type species *S. sanguineum* in the wild; however, the hypobasidia of the latter are solitary or in chains of 2–4, without a clamp connection at the base, and the clamp connection of hyphae is enlarged (5.5–8 μm). *S*. *jilinense* and *S*. *rubrofuscum* (Berk.) P. Roberts are similar; the basidiomata are all cerebriform and dark reddish brown, but they can be distinguished by the number of hypobasidia in the chains and the size of the basidiospores. Other species in *Sirobasidium* are white, off-white, or yellowish and are easily distinguished from *S. jilinense* (Table 2). Due to its brain-like, reddish-brown basidiomata, *S*. *jilinense* is easily confused with individual species of *Tremella* Pers. and *Exidia* Fr. in the wild, but it is easily distinguished from the latter under a microscope according to the basidium morphology.

Molecular phylogeny analysis showed that members of *Fibulobasidium* Bandoni and *Sirobasidium jilinense*, *S. magnum*, *S. apiculatum*, and *S. japonicum* form a separate branch (PP = 0.99, MLbs = 91%), while *S. brefeldianum* is located in another branch. This suggests that *Sirobasidium* is polyphyletic, which is consistent with the results of recent studies [9,10,11,13] (Figure 1). The new species *S. jilinense* and *S. magnum* have a sister relationship and obtained a high support rate (PP = 1, MLbs = 100%), which is consistent with the results of morphological studies.

*Calocera velutina* is characterized by basidiomata that are stipitate and pileate when young, needle-like when mature, yellow with a white disc-shaped villus at the base, and simple or dichotomously branched. The hyphae are thick-walled, without clamp connection. In summer, they grow on decaying trees in coniferous forests. In the wild, *C. velutina* is easy to be confused with *C. cornea* (Batsch) Fr., *C. sinensis* McNabb, and *C. tibetica* F. Wu, L.F. Fan & Y.C. Dai. They all have fresh yellow basidiomata with sharp, unbranched, or even-branched cylindrical upward-narrowing tops; however, the base of the latter three does not have a white disc-shaped villus [7,23,24]. The basidiomata of *C. sinensis* are small (1–5 mm), and the hyphae have clamp connections [23]. The basidiospores of *C. tibetica* are divided into three transverse septates when mature [24]. In the phylogenetic tree, they were also found to have a distant relationship.

*Dacrymyces jauensis* is characterized by basidiomata flat to cushion-shaped when young, cinnamon, golden yellow to brownish yellow; basidiospores sausage-shaped, separated horizontally when mature, usually divided into seven septate; germination produces rod-shaped conidia; hyphidia absent. *Dacrymyces jauensis*, *Dacrymyces san-augustinii* Kobayasi, and *Dacrymyces chiangraiensis* Ekanayaka, Karun., Q. Zhao & K.D. Hyde present multiple transverse septates when basidiospores mature. In the phylogenetic tree, a close relationship can be observed (Figure 2). However, the basidiomata of *Dacrymyces san-augustinii* are yellow, and the basidia (38–58 × 5.5–7 µm) and basidiospores (16–27.5 × 6–10 µm) are larger, with hyphidia [14]. The basidiomata of *Dacrymyces chiangraiensis* are yellow to orange, and the basidia (32–53 × 7–10 µm) and basidiospores (19–23 × 6.5–8 µm) are also large, with hyphidia [45].

There are two kinds of basidiomata of *Dacryopinax manghanensis*, most of which are clustered and spoon-shaped in the sand of the Inner Mongolia Autonomous Region but petal-shaped in Jilin Province (Figure 9). However, their microstructure is the same, as they are clustered in the same branch in the phylogenetic tree, and the shape of the basidiomata may be related to the environment. There are seven species of *Dacryopinax* distributed in China. The mature basidiospores of *Dacryopinax aurantiaca* and *Dacryopinax spathularia* are 0–1 septate, and they are easily confused with *Dacryopinax manghanensis* in the wild. However, the basidiospores of *Dacryopinax aurantiaca* are longer (10.4–13 × 3.5–5 μm) [7]. Basidiospores germinate as conidia or germ tubes and are mainly distributed in South China. *Dacryopinax spathularia* had obvious villi (visible to the naked eye when dry), with medulla on the transverse section and a bulbous hyphae septum [7]. In the phylogenetic tree, they are not closely related (Figure 2). In addition, the basidiospores of *Dacryopinax indacocheae* Lowy, *Dacryopinax primogenitus*, and *Dacryopinax sphenocarpa* Shirouzu & Tokum. are also divided into 0–1 septate at maturity. However, the basidiomata of *Dacryopinax indacocheae* are yellowish brown and larger (30 mm wide, 20 mm high). The cortical hyphae of the stipe have chain-like enlarged cells, the basidiospores are longer (8–11.5 × 3–3.5 μm), and the hymenium has abundant conidia [15]. *Dacryopinax primogenitus* has longer basidia (23–49 × 2.5–4 μm) and longer hyphidia (45.5–48.5 × 2.5–3 μm) and germinates as conidia or germ tubes [49]. The basidiomata of *Dacryopinax sphenocarpa* are yellow-white or light amber, the hyphae have clamp connections, the base of the probasidia has clamp connections, and the basidiospores are larger (10–16 × 4.5–8.5 μm) [14]. *Dacryopinax* is polyphyletic, with a total of eight species currently supported by molecular data, located in five distinct clades, consistent with recent findings [6,13,49]. In addition, in the phylogenetic tree constructed in this study, the branch composed of *Dacryopinax manghanensis* and *Dacryopinax* sp. (H7008759) was closely related to the branch composed of *Calocera* species (Figure 2); however, the species of *Dacryopinax manghanensis* and *Calocera* were different in morphology, so, it was placed in *Dacryopinax*.

Regarding jelly fungi, due to their gelatinous texture, researchers tend to focus more on damp environments when collecting specimens. In addition to adding three new species from the humid environment of Jilin Province—*S. jilinense*, *C. velutina*, and *Dacrymyces jauensis*—this study also added a new species from the Horqin Sandy Land—*Dacryopinax manghanensis*. This shows that jelly fungi have a wide geographical distribution and are well-adapted to different conditions and environments. As such, this type of fungus is worthy of further exploration.

## Figures and Tables

**Table 2:** Morphological comparison of *Sirobasidium* species.

Species	Basidiomata (mm)	Basidia	Basidiospores (μm)	Reference
*S. albidum*	circular, tremulous, albidus, 2–4	in chains of 8	fusiform, 24–26 × 6–10	[1]
*S. apiculatum*	pulvinate to cerebriform, white to grayish white, 2–4.5	2–cells	subglobose, 8.5–10.5 × 7.0–8.5	[13]
*S. brefeldianum*	droplet-like, white, 3	in chains of 10, 2 cells	globose 6–8	[2]
*S. japonicum*	pulvinate to applanate-cerebriform, white to pale yellow	in chains of 3–7, 4–cells	globose to subglobose, 4.0–7.0 × 3.5–6.5	[13]
*S. jilinense*	reddish brown to dark reddish brown, cerebriform, 1–3 cm	in chains of 3–9, 4–cells	subglobose to broadly ellipsoid, 6.7–9.4 × 6.7–7.2	This study
*S. minutum*	slight, pustular, white pink red	2–cells	globose, 5–5.4	[61]
*S. magnum*	cerebriform-vesicular flaps, yellow-brown to reddish brown, 8	in chains of 4–8, 2–4 cells	globose to subglobose, 6–9.5 × 6–9	[7]
*S. rubrofuscum*	pustular, cerebriform, dark reddish brown, 5–10	in chains of 2–4, 4–cells	subglobose, 7–7.5 × 8–8.5	[62]
*S. sandwicense*	hemispheric, albidus, 1–2	2–4 cells	subglobose, 6–9	[63]
*S. sanguineum*	gyroscopic-cerebriform, reddish, 4–20	in chains of 2–4, 4 cells	fusiform, 17–20 × 6–8	[1]

## Data Availability

All the sequences have been deposited in GenBank (https://www.ncbi.nlm.nih.gov, accessed on 1 May 2024) and MycoBank (https://www.mycobank.org, accessed on 17 May 2024). The data presented in this study are deposited in the Zenodo repository, accession number doi: 10.5281/zenodo.11206684.
